# Computational Approach to Investigating Key GO Terms and KEGG Pathways Associated with CNV

**DOI:** 10.1155/2018/8406857

**Published:** 2018-04-11

**Authors:** YuanYuan Luo, Yan Yan, Shiqi Zhang, Zhen Li

**Affiliations:** ^1^Department of Ophthalmology, School of Medicine, Renji Hospital, Shanghai Jiao Tong University, Shanghai 200127, China; ^2^Department of Biostatistics, University of Copenhagen, Copenhagen, Denmark

## Abstract

Choroidal neovascularization (CNV) is a severe eye disease that leads to blindness, especially in the elderly population. Various endogenous and exogenous regulatory factors promote its pathogenesis. However, the detailed molecular biological mechanisms of CNV have not been fully revealed. In this study, by using advanced computational tools, a number of key gene ontology (GO) terms and KEGG pathways were selected for CNV. A total of 29 validated genes associated with CNV and 17,639 nonvalidated genes were encoded based on the features derived from the GO terms and KEGG pathways by using the enrichment theory. The widely accepted feature selection method—maximum relevance and minimum redundancy (mRMR)—was applied to analyze and rank the features. An extensive literature review for the top 45 ranking features was conducted to confirm their close associations with CNV. Identifying the molecular biological mechanisms of CNV as described by the GO terms and KEGG pathways may contribute to improving the understanding of the pathogenesis of CNV.

## 1. Introduction

Choroidal neovascularization (CNV) is a serious eye disease involving the abnormal growth of blood vessels in the choroid region [1–3]. The growth originates from a break in Bruch's membrane, and subsequently the new blood vessels penetrate into the subretinal pigment epithelium [4]. From an epidemiological perspective, CNV is a major cause of pathological visual loss in aging populations [5]. Clinically, age-related macular degeneration (ARMD), myopia, and presumed ocular histoplasmosis syndrome (POHS) are the three major pathogeneses attributed to CNV [6–8]. The Wisconsin Beaver Dam Eye Study [9] confirmed that up to 90% of visual loss in ARMD is secondary to CNV. Given that ARMD is the most common cause of visual loss in people older than 50 years, CNV is speculated to be directly linked to such pathological visual loss. Aside from ARMD, the other two pathological processes—myopia [10] and POHS [11]—are also linked to pathological visual loss. This finding validates the specific role of CNV in pathological visual loss.

Clinically, most patients with CNV share a group of characteristic signs and symptoms, including painless loss of vision, metamorphopsia, paracentral or central scotoma, and apparent changes in image size perception [12, 13]. Generally, patients with these complaints need further physical examinations on blood, fluid, lipid exudation, and retinal pigment epithelial detachment for accurate diagnoses [14]. However, for the final differential diagnosis, laboratory tests are the golden criteria. Generally, the laboratory studies for CNV involve three main techniques, namely, fluorescein angiography [15], indocyanine green angiography [16], and spectral domain optical coherence tomography [17]. For the patients with confirmed diagnosis, due to the unclear pathological mechanisms of CNV, anti-VEGF treatment that counters angiogenesis is the only preferred clinical therapeutic approach [17]. However, the injection burden limits the long-term application of such anti-VEGF treatment. Therefore, more detailed pathological mechanisms of CNV need to be revealed to promote the development and application of new drugs against the disease.

Recent publications have partially revealed the detailed pathological mechanisms of CNV, which involve the interactions between genetic factors and exogenous environments. For the environmental factors, the personal physical factors induced by the exogenous factors are directly involved in the pathogenesis [18]. Age, obesity, high cholesterol, and high blood pressure aggravate the progression of CNV and further contribute to the occurrence of complications [19, 20]. Aside from these so-called physical exogenous factors, various genetic factors are also connected to the initiation and progression of CNV. Given that CNV is a highly specific disease with an abnormal angiogenesis, genes associated with angiogenesis, such as VEGF [21] and FGF2 [22], definitely participate in the pathological processes, which have been widely confirmed by reliable experiments. In addition to these genes, a specific gene called CFI participates in CNV and induces gradual visual loss and myopia; this finding is based on the sequencing data of CNV families [23]. Furthermore, a specific study [24] on the East Asian population with 2119 patients and 5691 controls revealed a group of effective hereditary and sporadic virulence genes that participate in CNV, mapping out the detailed genetic blueprint of CNV. Some trials were also conducted in the bioinformatics field. Zhang et al. [25] presented a specific computational routine for the identification of CNV-associated genes, indicating the efficacy and accuracy of computational application in such field.

As mentioned earlier, the genetic basis and the environmental influences of CNV have been revealed. However, its biological molecular mechanisms have not been explained thoroughly. Here, the detailed biological processes, cellular components, and molecular functions that may participate in the pathogenesis of CNV were screened out by using computational methods. In this study, GO [26] and KEGG [26, 27] pathways were introduced as two effective bioinformatics tools to accurately describe such items [27]. Based on widely known biological processes associated with CNV, an effective network was rebuilt, and novel biological processes described by the GO and KEGG items were screened out. Recent publications have validated these highly correlative biological processes, thus supporting the efficacy and accuracy of our prediction. With the use of computational methods, a group of functional biological processes that may participate in the potential pathogenesis of CNV were screened out, and for the first time, the detailed pathological mechanisms of CNV were described at the level of comprehensive biological processes instead of genes. The results contributed to the understanding of the development and progression of CNV.

## 2. Materials and Methods

**Figure 1:**
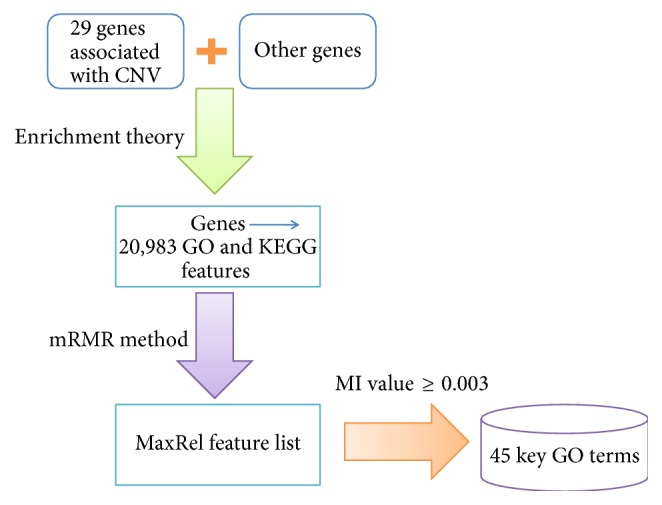
Flowchart of selecting the key GO terms and KEGG pathways related to CNV.

This study aimed to extract some key GO terms and KEGG pathways that share close biological associations with CNV by using a computational framework. The flowchart of our method is illustrated in Figure 1 for the easy understanding of this work.

### 2.1. Materials

In 2012, Newman et al. [28] reported a number of genes that are related to AMD. We downloaded the “Additional file 3” in their study [28], in which genes associated with AMD in literature either by genetic linkage or as expression biomarker were listed. Since CNV was a subtype of AMD, we further filtered the genes. Only the genes in CNV Up or CNV Down modules from “Additional file 5” in Newman et al.'s study were kept and at last, 35 CNV genes were obtained. CNV Up or CNV Down modules were generated by network clustering of differentially expressed genes with a permuted *p* < 0.1 and fold change *⩾* 1.5 among 31 normal, 7 MD1, 4 MD2, 17 Dry AMD, 2 GA, 4 CNV, and 3 GA/CNV samples. Therefore, the final CNV genes we used were both reported by literature and differentially expressed.

The obtained 35 CNV genes were mapped onto their Ensembl IDs. We excluded IDs that are not in the PPI network reported in STRING (Version 10.0) [29]. 38 Ensembl IDs were accessed. The GO terms and KEGG pathways were used to investigate the difference between CNV-related genes and others; thus, the Ensembl IDs without a GO term and KEGG pathway information were excluded. A total of 29 Ensembl IDs were left. These IDs were the positive samples in this study. The other 17,639 Ensembl IDs were the negative samples and comprised the dataset together with the positive samples in this study. The genes belonging to the positive and negative samples are provided in Supplementary Material S1.

### 2.2. Feature Vector

The goal of this study was to refine important GO terms and KEGG pathways that are associated with CNV genes. To fulfill that goal, all the genes in the dataset were needed to be represented by all the GO terms and KEGG pathways. Here, the enrichment theory [30] of the GO term and KEGG pathway was used to transform the genes into numeric values, which indicated the biological relationships between the genes and GO terms (KEGG pathways). Comparing with the direct binary annotation of whether a gene has a specific GO term or KEGG pathway, the score obtained by the enrichment theory can indicate the significance of overlap between a gene set and a GO or KEGG function in the genome background. It is more robust than the binary qualitative measurement [31]. To date, this theory has been widely applied to investigate different gene- or protein-related problems [30, 32–41]. After each gene was represented by a larger number of features, by applying a feature selection method described in Section 2.3, the key GO term or KEGG pathway features were extracted to distinguish the difference between the positive and negative samples. The encoding procedure follows.

*GO Enrichment Score*. The GO enrichment score was utilized to represent the association between a GO term and an involved gene as a numeric value. For a given GO term, such as GO_*j*_, and a gene *g*, the gene set *G*_1_ consisted of genes annotated to GO_*j*_ and gene set *G*_2_ consisted of the neighbor genes of *g* in the protein–protein interaction network reported in STRING (http://string-db.org/) [29], a well-organized database providing known and predicted protein–protein interactions. On the basis of the preceding items, the GO enrichment score of GO_*j*_ and *g* can be defined as the −log_10_ of the hypergeometric test *p* value [30, 32–35] of *G*_1_ and *G*_2_ according to the following equation:

(1) $$\begin{array}{c} ES_{GO}\left(\;g,GO_{j}\;\right)=-log_{10}\left(\;\overset{n}{\underset{k=m}{\sum }}\frac{\left(\begin{array}{c} M\\ k \end{array}\right)\left(\begin{array}{c} N-M\\ n-k \end{array}\right)}{\left(\begin{array}{c} N\\ n \end{array}\right)}\;\right), \end{array}$$

where *N* is the total number of genes in humans, *M* is the number of genes in *G*_1_, *n* is the number of genes in *G*_2_, and *m* is the number of the common genes of *G*_1_ and *G*_2_. A large enrichment score of GO_*j*_ and *g* indicated a close relationship between them. In this study, 20,686 GO terms were considered. Thus, 20,686 GO enrichment scores were calculated for each gene in the dataset, which were obtained by using an in-house program using R function phyper. The R code is “score *←* −log10(phyper(numWdrawn − 1, numW, numB, numDrawn, lower.tail = FALSE)),” where numW, numB, and numDrawn correspond to the number of genes annotated to GO_*j*_, number of genes not annotated to GO_*j*_, and number of neighbor genes *g*, respectively. 

*KEGG Enrichment Score*. Similar to the GO enrichment score, the KEGG pathway score was calculated using the same theory to represent the quantitative associations between the KEGG pathways and genes in the dataset. For a given KEGG pathway *K*_*j*_ and a gene *g*, *G*_1_ was a gene set containing genes in *K*_*j*_ and *G*_2_ had the same meaning as described in preceding paragraph. The KEGG enrichment score shared a similar definition with the GO enrichment score between *K*_*j*_ and *g*, which was formulated as

(2) $$\begin{array}{c} ES_{KEGG}\left(\;g,K_{j}\;\right)=-log_{10}\left(\;\overset{n}{\underset{k=m}{\sum }}\frac{\left(\begin{array}{c} M\\ k \end{array}\right)\left(\begin{array}{c} N-M\\ n-k \end{array}\right)}{\left(\begin{array}{c} N\\ n \end{array}\right)}\;\right), \end{array}$$

where *N*, *M*, *n*, and *m* share similar definitions as described in (1). In addition, a high score yielded by a KEGG pathway *K*_*j*_ and a gene *g* indicated their strong associations. Here, 297 KEGG pathways were considered and resulted in 297 KEGG enrichment scores for each gene, which were also obtained by using an in-house program using R function phyper.

Accordingly, each gene in the dataset was encoded by a combination of 20,686 GO term and 297 KEGG pathway features and was defined as a feature vector with a total of 20,983 elements:

(3) fg=ESGOg,GO1,…,ESGOg,GO20686,ESKEGGg,K1,…,ESKEGGg,K291T.

### 2.3. Feature Selection

As described in Section 2.2, each gene in the dataset was encoded with 20,983 features derived from the GO terms and KEGG pathways. Some of them shared closer biological associations with CNV. Thus, advanced tools were necessary to extract these important features that played essential roles in the development of CNV. Here, a reliable and widely accepted feature selection method, namely, maximum relevance and minimum redundancy (mRMR) [42], was adopted to analyze all 20,983 features. The mRMR method, proposed by Peng et al. [42], is a useful tool to analyze the feature space of complicated biological problems. To date, many investigations related to complicated biological systems or problems have applied this method to analyze their feature space and extract important information [34, 36, 43–52].

In the mRMR method, two excellent criteria were proposed to rank the features: (1) maximum relevance and (2) minimum redundancy. According to their names, the former criterion measures the importance of features by relying on their correlation to target variable, whereas the latter criterion provides a guarantee that the selected features also have minimum redundancies. If one decides to construct an optimal feature subspace, both maximum relevance and minimum redundancy should be used. In this study, the purpose was to extract important features that are closely related to CNV rather than construct an optimal feature subspace. Therefore, only the criterion of maximum relevance was employed to rank the features in this study. The maximum relevance of each feature was measured by the mutual information (MI) between the feature and the target variable. For each feature, *f* was a variable representing the values in all samples and *c* was the target variable. The MI was calculated as follows:

(4) $$\begin{array}{c} I\left(\;c,f\;\right)=\iint p\left(\;c,f\;\right)\log\frac{p\left(c,f\right)}{p\left(c\right)p\left(f\right)}dc\;df, \end{array}$$

where *p*(*c*) and *p*(*f*) are the marginal probabilities of *c* and *f* and *p*(*c*, *f*) is their joint probabilistic distribution. According to (4), MI measures the mutual dependence between two variables.

Based on the MI value assigned to each feature, the feature ranking list called MaxRel feature list was obtained and formulated as follows:

(5) $$\begin{array}{c} F=\left[\;f_{1},f_{2},\ldots ,f_{N}\;\right], \end{array}$$

where *N* is the total number of features in the feature space. A high rank received by a feature indicates a strong association with CNV. Based on the properties of the top ranked features, a new insight into the CNV can be proposed for the investigation of the corresponding GO terms and KEGG pathways.

## 3. Results and Discussion

### 3.1. Results

As described in Section 2.2, a total of 20,983 GO terms and KEGG pathway features were encoded in each gene in dataset. Then, according to their relevance to the target variables, these features were ranked in the descending order by using the maximum relevance criterion described in Section 2.3. The output feature list, called MaxRel feature list, was built and obtained (Supplementary Material S2).

**Figure 2:**
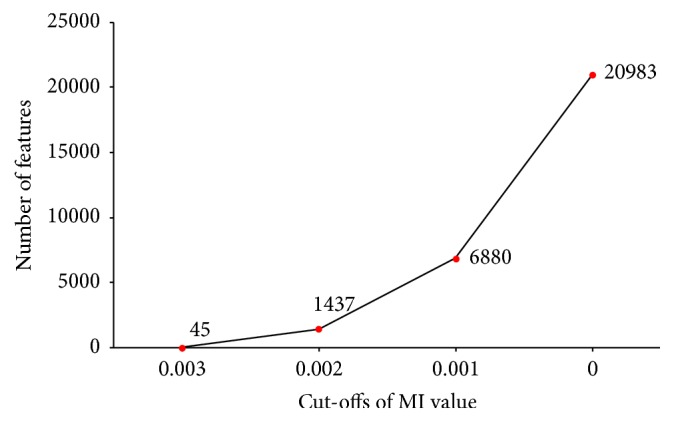
The number of selected features under different cut-offs of MI values.

As mentioned in the preceding paragraphs, not all GO terms or KEGG pathways shared equal roles on influencing the progression of CNV. Thus, extracting the key GO terms or KEGG pathways was necessary. By applying the maximum relevance criterion, all the features were ranked by their relevance to the target variables, and the rank of a corresponding feature in the output MaxRel feature list for a GO term or KEGG pathway indicated its association with CNV. According to their MI values, some GO terms or KEGG pathways received high MI values in the MaxRel feature list; these features were extracted and their importance was further investigated. To determine the cut-off of MI value, a curve was plotted in Figure 2, which shows the number of selected features under different cut-offs of MI value. It can be observed that the cut-off 0.003 was a proper choice, resulting in 45 features. These features would be given further literature review. Their detailed information is listed in Table 1. All the 45 features corresponded to important GO terms. The following section provides a detailed discussion on these GO terms.

### 3.2. Analysis of Key GO Terms

As mentioned earlier, based on our current computational methods, a group of functional biological processes that may directly contribute to the initiation and progression of CNV as a pathological mechanism were screened out. In the prediction list, the top 45 biological processes described by the GO terms as optimal CNV-associated biological processes were selected. Due to the limitation of such manuscript, an individual analysis of all the items was not feasible. Therefore, the top terms were chosen, and their respective connection with CNV according to recent publications was discussed. According to recent publications, such GO terms can be summarized into three major subgroups: angiogenesis, local neural metabolism, and immune-associated biological processes. The detailed discussion follows.

#### 3.2.1. Analysis of Angiogenesis Associated Biological Processes

The two GO terms in our prediction list—*GO: 0031091* and* GO: 0031093*—both describe the functional cellular components of platelet alpha-granules. In 2015, a specific study [53] on proangiogenic responses confirmed that the release of platelet alpha-granules promotes angiogenesis. No direct connections were revealed between platelet alpha-granules and CNV; however, abnormal angiogenesis plays an irreplaceable role and may be the core pathological biological process during the initiation and progression of CNV [54]. Therefore, GO items associated with platelet alpha-granules, such as cellular components GO: 0031091 and GO: 0031093, are definitely associated with CNV. This result validated the efficacy and accuracy of our prediction.

GO term* GO: 0038133 *describes a detailed pathway called the ERBB2-ERBB3 signaling pathway. According to recent publications, this signaling pathway contributes to the regulation of cell survival and tumorigenesis [55, 56]. As for the detailed connections between the ERBB2-ERBB3 signaling pathway and CNV, mediated by miR-199a and miR-125b, ERBB2 and ERBB3 as two functional components of our predicted biological process have been confirmed to contribute to the regulation of vascular endothelial growth factor secretion and the stimulation of angiogenesis in multiple tissues, including the eyes [57–59]. Given the core initiative functions of angiogenesis for CNV, the predicted biological process called the ERBB2-ERBB3 signaling pathway is a potential CNV-associated GO term. Moreover, the next predicted GO term, called* GO: 0038129*, also describes the ERBB3-associated signaling pathway. This finding not only implied the prediction consistency of the current computational methods but also further confirmed the specific role of such pathways during the initiation and progression of CNV.

*GO: 0031983 *was the next predicted GO term and describes the vesicle lumen as a functional cellular component. As the parent term of GO: 0060205 describing the cytoplasmic vesicle lumen, such cellular component definitely is associated with the initiation and progression of CNV. As for detailed literature evidence, in 2009, a specific study on the vascular permeability and pathological angiogenesis of CNV confirmed that the vesicle lumen in living cells is related to the vascular hyperpermeability and abnormal angiogenesis [60]. Vascular hyperpermeability [61] and abnormal angiogenesis are both specific symptoms of CNV [62]; thus, such biological processes are potential CNV-associated biological processes.

The next GO term, called* GO: 0005576*, describes a general term called extracellular region. Various extracellular substances participate in the pathogenesis of CNV, including LOX [63], LOXL2 [63], Thy-1 [64], and integrins [65]. Such specific substances may play irreplaceable roles during the initiation and progression of CNV; thus, this GO term that describes the extracellular regions of a certain focus is a potential CNV-associated biological process.* GO: 0035767*, as the next predicted GO in our prediction list, describes an effective biological process called endothelial cell chemotaxis. Based on recent publications, such biological process is involved in the activation of platelets [66] and exosome-mediated antiangiogenesis [67]. Platelet activation [68] and angiogenesis [69] are directly connected to the initiation and progression of CNV; therefore, this predicted GO term is quite significant in the pathogenesis of CNV.

#### 3.2.2. Analysis of Local Neural Metabolism Associated Biological Processes

*GO: 0060205*, as another cellular component associated item, describes the cytoplasmic vesicle lumen. Based on recent publications, such cellular component participates in autophagy and secretion-associated biological processes in living cells [70, 71]. As for the biological connections between the cytoplasmic vesicle lumen and CNV, the predicted GO-associated biological processes, such as autophagy and substance secretion, have all been confirmed to be involved in the initiation and progression of CNV [72, 73]. This result implied the accuracy and efficacy of our prediction.* GO: 1902847* describes the regulation of neuronal signal transduction. In the biological process of neuronal signal transduction, a specific gene called IKK2 has been confirmed to be significant [74]. Coincidentally, the inhibition of IKK2 has been widely used in the treatment against CNV, indicating the specific role of IKK2 during the pathogenesis of CNV [75, 76]. Therefore, connected by such functional gene IKK2, the predicted biological processes associated with neuronal signal transduction may also be related to CNV. This finding validates the efficacy and accuracy of our prediction. As the next predicted GO,* GO: 1902949* describes the positive regulation of tau protein kinase activity. Tau protein is a major pathological factor that contributes to the initiation and progression of Alzheimer's disease (AD) [77–79]. During the initiation and progression of AD, another specific protein called apolipoprotein E4 (apoE4) interacts with our predicted tau protein [80] and participates in the pathogenesis of AD [81]. Given that recent studies also validated the specific role of apoE4 in neovascularization [82] and its potential functions in CNV [23], tau protein associated kinase activity is reasonably connected to CNV-associated biological characteristics.

#### 3.2.3. Analysis of Immune-Associated Biological Processes

The GO term* GO: 0061517* describes the proliferation of a specific immune-associated cell subgroup: macrophage. Based on recent publications, macrophages contribute to CNV by regulating CCR2-dependent and proangiogenic biological processes [83, 84], indicating that the proliferation of such gene is definitely related to the progression of the disease [85]. Apart from the proliferation of macrophage, the proliferation of another effective cell subgroup called microglial cells is also predicted to contribute to CNV by* GO: 0061518* in our prediction list. Mediated by neuroprotectin D1, microglial ramifications and redistribution participate in the pathological processes of CNV [86]. Therefore, as a functional neuronal cell subtype with specific microglial ramifications [86], this predicted GO is reasonably connected to CNV [87]. Besides these predicted biological processes, several functional GOs in the top 45 predicted GO terms have been reported to participate in CNV-associated biological processes. These functional GO terms include GO: 0002580 (regulation of antigen processing and presentation of peptide or polysaccharide antigen via MHC class II) [88], GO: 0044421 (extracellular region) [89], and GO: 0007603 (phototransduction, visible light) [90, 91]. These results confirmed the efficacy and accuracy of our prediction.

## 4. Conclusion

Based on our presented computational method, a group of functional biological functions that have been confirmed by recent publications to be related to the pathogenesis of CNV were screened out. Such predicted biological processes not only further revealed the detailed pathological mechanisms of CNV but also provided a new tool to identify potential functional disease-associated biological processes in multiple categories of the disease. Finally, we will try our best to develop a computational method based on some extracted features in this study to predict novel CNV genes in the future.

## Figures and Tables

**Table 1:** Top 45 key GO terms associated with CNV.

GO term ID	GO term	GO description	MI value	Rank
GO: 0031091	Platelet alpha-granule	Cellular component	0.003	1
GO: 0031093	Platelet alpha-granule lumen	Cellular component	0.003	2
GO: 0060205	Cytoplasmic membrane-bounded vesicle lumen	Cellular component	0.003	3
GO: 0038133	ERBB2-ERBB3 signaling pathway	Biological process	0.003	4
GO: 0038129	ERBB3 signaling pathway	Biological process	0.003	5
GO: 1902847	Regulation of neuronal signal transduction	Biological process	0.003	6
GO: 0061517	Macrophage proliferation	Biological process	0.003	7
GO: 1902949	Positive regulation of tau protein kinase activity	Biological process	0.003	8
GO: 0061518	Microglial cell proliferation	Biological process	0.003	9
GO: 0031983	Vesicle lumen	Cellular component	0.003	10
GO: 0005576	Extracellular region	Cellular component	0.003	11
GO: 0035767	Endothelial cell chemotaxis	Biological process	0.003	12
GO: 0002580	Regulation of antigen processing and presentation of peptide or polysaccharide antigen via MHC class II	Biological process	0.003	13
GO: 0044421	Extracellular region part	Cellular component	0.003	14
GO: 0007603	Phototransduction, visible light	Biological process	0.003	15
GO: 0001948	Glycoprotein binding	Molecular function	0.003	16
GO: 0072562	Blood microparticle	Cellular component	0.003	17
GO: 0044650	Adhesion of symbiont to host cell	Biological process	0.003	18
GO: 0019062	Virion attachment to host cell	Biological process	0.003	19
GO: 0010466	Negative regulation of peptidase activity	Biological process	0.003	20
GO: 0009584	Detection of visible light	Biological process	0.003	21
GO: 0010951	Negative regulation of endopeptidase activity	Biological process	0.003	22
GO: 0001654	Eye development	Biological process	0.003	23
GO: 0002581	Negative regulation of antigen processing and presentation of peptide or polysaccharide antigen via MHC class II	Biological process	0.003	24
GO: 0052547	Regulation of peptidase activity	Biological process	0.003	25
GO: 0052548	Regulation of endopeptidase activity	Biological process	0.003	26
GO: 0005791	Rough endoplasmic reticulum	Cellular component	0.003	27
GO: 0050839	Cell adhesion molecule binding	Molecular function	0.003	28
GO: 0071307	Cellular response to vitamin K	Biological process	0.003	29
GO: 1902430	Negative regulation of beta-amyloid formation	Biological process	0.003	30
GO: 0005604	Basement membrane	Cellular component	0.003	31
GO: 0030023	Extracellular matrix constituent conferring elasticity	Molecular function	0.003	32
GO: 2000768	Positive regulation of nephron tubule epithelial cell differentiation	Biological process	0.003	33
GO: 1903002	Positive regulation of lipid transport across blood brain barrier	Biological process	0.003	34
GO: 1903000	Regulation of lipid transport across blood brain barrier	Biological process	0.003	35
GO: 1903001	Negative regulation of lipid transport across blood brain barrier	Biological process	0.003	36
GO: 1902951	Negative regulation of dendritic spine maintenance	Biological process	0.003	37
GO: 1902999	Negative regulation of phospholipid efflux	Biological process	0.003	38
GO: 1901627	Negative regulation of postsynaptic membrane organization	Biological process	0.003	39
GO: 2001139	Negative regulation of phospholipid transport	Biological process	0.003	40
GO: 0046911	Metal chelating activity	Molecular function	0.003	41
GO: 0030574	Collagen catabolic process	Biological process	0.003	42
GO: 0007423	Sensory organ development	Biological process	0.003	43
GO: 0001968	Fibronectin binding	Molecular function	0.003	44
GO: 0051346	Negative regulation of hydrolase activity	Biological process	0.003	45

## Supplementary Materials

Supplementary Materials The supplementary materials consist of two files. Supplementary Material S1: positive and negative samples in the dataset represented by their gene symbols. Supplementary Material S2: MaxRel feature list as an output of the mRMR method with ranked 20,983 features.
